# Theoretical Investigation of Competitive Adsorption of Light and Heavy Rare Earth Ions on the (001) Surface of Kaolinite

**DOI:** 10.3390/molecules30040838

**Published:** 2025-02-11

**Authors:** Sen Qiu, Yijin Hua, Zehao Fan, Qibang Long, Kuifang Zhang, Xuwei Lian, Tao Tu, Li Li, Tingsheng Qiu

**Affiliations:** 1 School of Intelligent Manufacturing and Materials Engineering, Gannan University of Science and Technology, Ganzhou 341000, China; hxqiusen@163.com (S.Q.); y771h15@163.com (Y.H.); zhang_kui_fang@163.com (K.Z.); lxwyui@163.com (X.L.); 9320040150@gnust.edu.cn (T.T.); lilihg982@163.com (L.L.); 2 Key Laboratory of Ionic Rare Earth Resources and Environment, Ministry of Natural Resources of the People’s Republic of China, Ganzhou 341000, China; 3 College of Resource and Environmental Engineering, Jiangxi University of Science and Technology, Ganzhou 341000, China; fzh15180177986@163.com (Z.F.); lqb112358@163.com (Q.L.)

**Keywords:** rare earth, kaolinite, adsorption, molecular dynamics

## Abstract

Kaolinite is the primary mineral in ion-adsorption rare earth ores, and improving our understanding of the adsorption state of REEs on kaolinite will benefit efforts to leach REEs from these ores. In order to explain why Eu^3+^ ions exhibit stronger pH-dependent desorption behavior than Lu^3+^, molecular dynamics simulations were applied to investigate the adsorption mechanism of Eu^3+^ and Lu^3+^ on a deprotonated kaolinite (001) surface. The simulation results show that the hydration numbers of Eu^3+^ and Lu^3+^ are comparable, whereas the ordering degree of water molecules around Eu^3+^ is higher than that of Lu^3+^, which is beneficial to the movement of Eu^3+^ to preferentially occupy favorable adsorption sites on the kaolinite surface, following which coordination bonds are formed between Eu^3+^ and the surface. As a result, the desorption rate of Eu^3+^ decreases sharply with the increase in leaching pH, while the desorption rate of Lu^3+^ is only slightly affected by pH.

## 1. Introduction

Rare earth elements (REEs) are known as industrial vitamins due to their unique physical and chemical properties; thus, they are widely applied in the fields of magnetism, nuclear energy, catalysis, and medicine [1,2]. There are two different kinds of RE ores, depending on the existence state of REEs [3]: mineral-type and ion-adsorption RE ores. Mineral TRE ores, such as bastnaesite and monazite, mainly contain light REEs (La→Gd), while heavy REEs (Tb→Lu + Y) are only present in ion-adsorption RE ores [4]. The value of heavy REEs is much higher than that of light REEs. Ion-adsorption RE ores are only present in southeast Asia, with the REEs mainly adsorbed on clay mineral surfaces (e.g., kaolinite) in the forms of hydrated ions or hydroxyl-hydrated ions, meaning they can be desorbed through ion exchange with concentrated inorganic salts such as NH_4_^+^, Mg^2+^, and Al^3+^ [5,6,7].

Kaolinite is a typical 1:1 lamellar silicate mineral, with a basic unit of Si-O-Al-O-H [8]. This basic unit usually cleaves the (001) surface, and, subsequently, the Si-O and Al-OH surfaces are exposed, which is beneficial for the adsorption of metal ions. In addition, there are permanent and variable pH-dependent charges on the kaolinite’s surface due to isomorphous substitution and protonation/deprotonation of amphoteric hydroxyl groups, respectively [9,10,11]. As a result, RE ions can be adsorbed through electrostatic attraction and surface complexation [12].

The adsorption/desorption behaviors of RE ions on kaolinite have been widely investigated because kaolinite is the primary mineral carrier of REEs in ion-adsorption RE ores. Moldoveanu et al. [13,14] investigated the leaching efficiency of cations (Li^+^, Na^+^, Cs^+^, and NH_4_^+^) and anions (SO_4_^2−^ and Cl^−^) for RE ions from clay minerals of ion-adsorption RE ores, revealing that Cs^+^ > NH_4_^+^ > Na^+^ > Li^+^ and SO_4_^2−^ > Cl^−^. Xiao et al. [15] revealed that the adsorption capacities of La, Nd, and Y on the surface of kaolinite are 1.731, 1.587, and 0.971 mg/g, respectively, showing that kaolinite prefers to adsorb light REEs. In addition, they also found that with the increase in the depth of the ion-adsorption RE ore body, the amount of light REEs gradually decreased, while the amount of heavy REEs showed an upward trend. He et al. [16] found that light RE ions are preferentially adsorbed by kaolinite at high ionic strengths, and that the fractionation effect of RE ions in ion-adsorption RE ores is mainly due to selective adsorption of light RE ions by kaolinite and halloysite.

The above studies mainly concentrated on experimental phenomena, while little attention has been paid to adsorption/desorption mechanisms. Chemical theoretical calculations are an effective method to investigate the reaction mechanisms on molecular and atomic scales [17,18,19]. In previous studies, we found that hydrated RE ions can be adsorbed on the kaolinite’s Si-O surface through hydrogen bonds, while coordination and hydrogen bonds were found on the Al-OH surface. The hydroxyl groups of the Al-OH surface can be protonated and deprotonated, and because active reactions of the surface are enhanced when the surface hydroxyl is deprotonated, coordination bonds form between hydrated RE ions and deprotonated oxygen [20,21]. A similar conclusion was also found by Peng [22] and Wang et al. [23], who revealed that the adsorption energy of RE ions increased with the increase in the degree of surface hydroxyl deprotonation. All of these works investigated the adsorption of single hydrated RE ions on kaolinite using density functional theory (DFT) and made lateral comparisons, ignoring the competitive adsorption between light and heavy RE ions. Molecular dynamics (MD) calculations can simulate systems that resemble the real reaction environment to obtain statistical parameters that supplement DFT calculations [24,25].

In our previous work [26], we found clear differences in desorption behavior between light and heavy REEs; however, the mechanism behind these differences is unclear. Hence, the competitive adsorption behavior of light and heavy REEs on kaolinite surfaces is studied through this experiment. Moreover, MD calculations were used to reveal the microscopic mechanisms of competitive adsorption at the molecular level. This research is beneficial to enriching metallogenic theory and optimizing leaching technology for ion-adsorption RE ores.

## 2. Results and Discussion

### 2.1. Experimental Results

#### 2.1.1. Desorption Characteristics of Eu^3+^ and Lu^3+^ from Kaolinite

**Figure 1:**
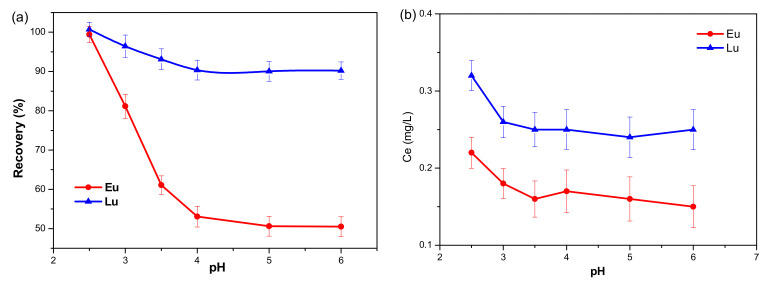
Effect of pH on the recovery of Eu^3+^ and Lu^3+^ from adsorbed (**a**) and raw (**b**) kaolinite.

The recovery of Eu^3+^ and Lu^3+^ from adsorbed and raw kaolinite was evaluated as a function of the pH value. As shown in Figure 1a, the desorption characteristics of Eu^3+^ and Lu^3+^ from adsorbed kaolinite were clearly different. The recovery of Eu^3+^ first decreased sharply with an increase in pH, and then remained constant at 50.5% when the pH was higher than 4. By contrast, the recovery of Lu^3+^ decreased slightly with an increase in pH, but a recovery as high as 90.2% was observed when the pH was lowered to 6. In Figure 1b, the equilibrium concentrations of Eu^3+^ and Lu^3+^ are 0.23 mg/L and 0.32 mg/L, respectively, which are much lower than the equilibrium concentrations of Eu^3+^ (21.1 mg/L) and Lu^3+^ (25.2 mg/L) desorbed from kaolinite. These results demonstrate that the Eu^3+^ and Lu^3+^ released from kaolinite almost always originate from adsorption.

Previous studies have shown that RE ions are adsorbed on clay surfaces through cation exchange, surface complexes, and electrostatic adsorption [27,28]. In addition, RE ions tend to adsorb on the deprotonated kaolinite surface through coordination bonds, which are hard to remove. Therefore, the low recovery of Eu^3+^ at a high pH might due to the coordination bonds between Eu^3+^ and the deprotonated surface. This observation can be verified by the following MD simulations. In order to clearly reveal the differences in desorption characteristics between Eu^3+^ and Lu^3+^, the competitive kinetics of adsorption of Eu^3+^ and Lu^3+^ onto kaolinite were studied.

#### 2.1.2. Adsorption Kinetics

**Figure 2:**
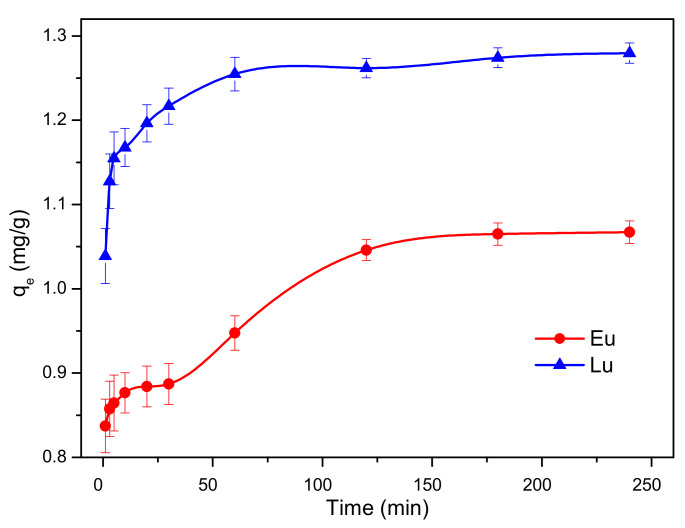
Effect of contact time on Eu^3+^ and Lu^3+^ adsorption onto kaolinite.

The competitive adsorption efficiencies of Eu^3+^ and Lu^3+^ on kaolinite are shown in Figure 2. The q_e_ of Lu^3+^ was slightly higher than that of Eu^3+^, indicating that the fractionation effect for light and heavy REEs occurred on kaolinite. The q_e_ of Eu^3+^ and Lu^3+^ increased with an increase in contact time, and adsorption equilibria were reached at 120 min and 60 min, respectively. As the contact time continued to increase, there was little increase in the adsorption capacity; however, 95% of Lu^3+^ was adsorbed within 20 min, while only 82% of Eu^3+^ was adsorbed. These results imply that the adsorption rate of Eu^3+^ was much slower than that of Lu^3+^, which might be due to the presence of a different adsorption mechanism. The slow adsorption rate of Eu^3+^ might be due to the formation of coordination bonds between Eu^3+^ and the kaolinite surface, which leads to low desorption rates at high pH values. Moreover, the adsorption of almost all of Lu^3+^ was quite rapid, which might be due to the weak electrostatic attraction between Lu^3+^ and the surface [20]. Hence, Lu^3+^ was easily desorbed at high pH values. Eu^3+^, which is more firmly attached to kaolinite, required lower pH values for full desorption [29].

Pseudo-first-order [30] and pseudo-second-order [31] models were adopted to study the kinetic adsorption characteristics of Eu^3+^ and Lu^3+^ on kaolinite. The corresponding models are expressed in Equations (1) and (2).

The pseudo-first-order model is

(1) $$\ln\left(q_{e}-q_{t}\right)=lnq_{e}-k_{1}t$$

The pseudo-second-order model is

(2) $$\frac{t}{q_{t}}=\frac{1}{q_{e}}t+\frac{1}{k_{2}q_{e}^{2}}$$

where *q_t_* is the adsorption capacity of RE ions at time *t*, and *k*_1_ and *k*_2_ are the rate constants for pseudo-first-order and pseudo-second-order models, respectively.

**Figure 3:**
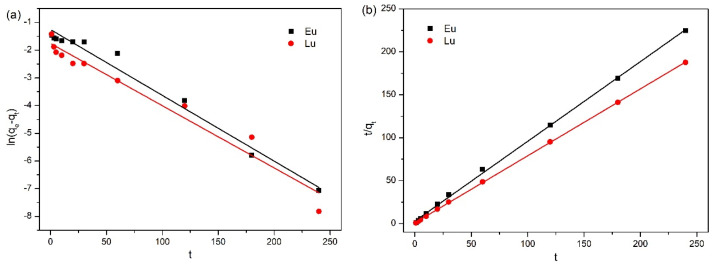
Pseudo-first-order (**a**) and pseudo-second-order (**b**) for Eu^3+^ and Lu^3+^ onto kaolinite.

The fitted results of the kinetics of Eu^3+^ and Lu^3+^ adsorption on kaolinite are shown in Figure 3, while Table 1 lists the values of kinetics parameters for pseudo-first-order and pseudo-second-order models. The correlation coefficient values (R^2^) of Eu^3+^ and Lu^3+^ for pseudo-second-order models were 0.998 and 0.999, respectively, which are higher than those of pseudo-first-order models, suggesting that the adsorption kinetics of Eu^3+^ and Lu^3+^ on kaolinite all matched well to the pseudo-second-order model; a similar conclusion was obtained by Guan et al. [32]. In addition, the larger *k_2_* of Lu^3+^ implies that the adsorption rate of Lu^3+^ was higher than that of Eu^3+^.

#### 2.1.3. Adsorption Isotherms

**Figure 4:**
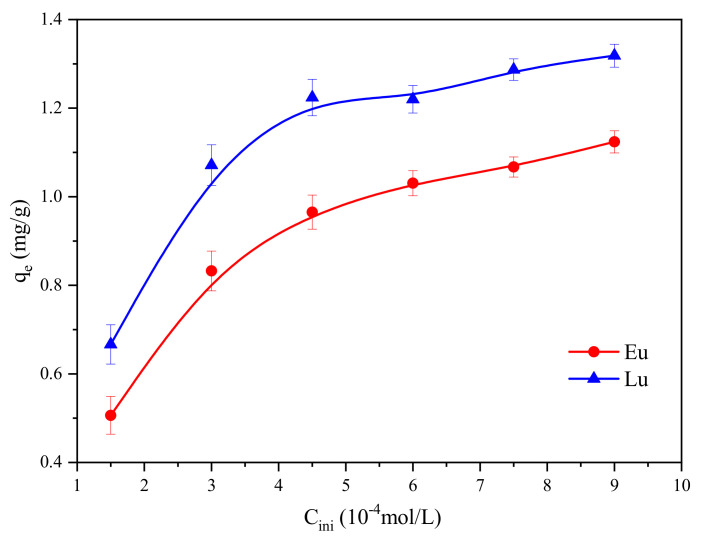
The adsorption isotherms of Eu^3+^ and Lu^3+^ onto kaolinite.

The effect of different initial concentrations of Eu^3+^ and Lu^3+^ on the equilibrium adsorption capacities are presented in Figure 4. The adsorption capacities of Eu^3+^ and Lu^3+^ increased rapidly with the increase in initial concentrations, but increased slowly when initial concentrations were higher than 0.45 mmol/L. The results indicated that the increase in initial concentration of Eu^3+^ and Lu^3+^ was beneficial to the increase in adsorption capacities, since the increase in initial Eu^3+^ and Lu^3+^ concentrations increases the mass-transfer driving force of Eu^3+^ and Lu^3+^ between the solution and the kaolinite surface [33].

Many isotherms have been used to model equilibrium adsorption data to obtain adsorption information such as adsorption mechanisms, maximum adsorption capacities, and adsorbent properties [34]. In this study, two-parameter adsorption isotherms (the Langmuir and Freundlich model) and three-parameter adsorption isotherms (the Langmuir–Freundlich model) were chosen to describe the properties of the adsorption of Eu^3+^ and Lu^3+^ on the kaolinite surface. Since linearization of the adsorption model changes the dependent and independent variables and introduces propagation errors [34,35], a nonlinear fitting method was chosen for this study.

The basic assumptions of the Langmuir isotherm are [34] as follows: (1) monolayer adsorption; (2) homogenous distribution of adsorption sites; (3) constant adsorption energy; and (4) a negligible interaction between adsorbate molecules. The nonlinear Langmuir model is presented as follows:

(3) $$Q_{e}=\frac{Q_{L}K_{L}C_{e}}{1+K_{L}C_{e}}$$

where *Q_e_* is the equilibrium adsorption capacity (mg/g), *K_L_* is the Langmuir constant (L/mg), *C_e_* is the equilibrium concentration (mg/L), and *Q_L_* is the maximum adsorption capacity (mg/g).

The Freundlich model [36] assumes the heterogeneity of the surface, as well as that adsorption occurs at different active sites. Stronger binding sites are occupied first, and the binding strength decreases with an increase in site occupation [37]. The nonlinear Freundlich model is presented as follows:

(4) $$Q_{e}=K_{F}C_{e}^{n_{F}}$$

where *Q_e_* is the equilibrium adsorption capacity (mg/g), *K_F_* is the Freundlich adsorbent capacity ${\left(mg/g)/(mg/L\right)}^{n_{F}}$, and *n_F_* is a constant.

However, the adsorption process is often complex: the adsorbent surface is not completely homogeneous, adsorption sites are inhomogeneous, and the adsorption process may also coexist with chemisorption and physisorption. Due to this, the Langmuir and Freundlich adsorption isotherm models have some limitations, and the latter is also unable to determine the maximum adsorption capacity. On this basis, the Langmuir–Freundlich isotherm model was developed by combining the two isotherm models [38,39,40]. The Langmuir–Freundlich isotherm model assumes that at low adsorbate concentrations, the model satisfies the Freundlich isotherm model, whereas under high-adsorbate-concentration conditions, it will satisfy the Langmuir isotherm model [41]. Its nonlinear equations are expressed as follows:

(5) $$Q_{e}=\frac{Q_{LF}{\left(K_{LF}C_{e}\right)}^{n_{LF}}}{1+{\left(K_{LF}C_{e}\right)}^{n_{LF}}}$$

where *Q_e_* is the equilibrium adsorption capacity (mg/g), *Q_LF_* (mg/g) is the Langmuir–Freundlich maximum adsorption capacity of the adsorbent; *K_LF_* (L/mg) is the Langmuir–Freundlich equilibrium constant; and *n_LF_* (dimensionless) is the exponent of the Langmuir–Freundlich model.

**Figure 5:**
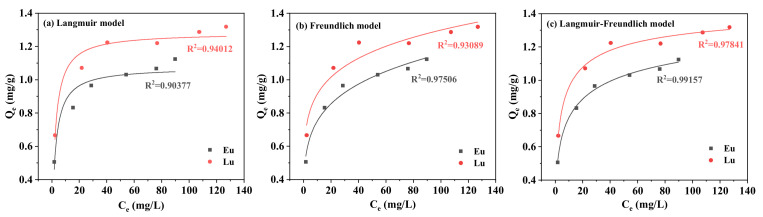
The nonlinearized Langmuir (**a**), Freundlich (**b**), and Langmuir-Freundlich isotherm (**c**) for Eu^3+^ and Lu^3+^ onto kaolinite.

The fitting plots of Langmuir, Freundlich, and Langmuir–Freundlich adsorption isotherm models of Eu^3+^ and Lu^3+^ are shown in Figure 5, and Table 2 presents the corresponding parameters of these models. As the adjusted correlation coefficients (adj-R^2^) are above 0.90, it can be concluded that almost all selected models fit the experimental data of the adsorption process well. A higher correlation coefficient (R^2^ >0.97) and a lower red-χ^2^ (reduced chi-square statistic) value for the Langmuir–Freundlich model suggest that the sorption isotherms were better fitted by this model than by the Langmuir and Freundlich models. In addition, the Langmuir–Freundlich isotherm model overestimates the value of maximum adsorption, which has also been observed in other studies [40]. Based on the basic assumptions of the Langmuir–Freundlich isotherm model, the following conclusions are drawn: Both homogeneous and heterogeneous active adsorption sites are present on the surface of kaolinite, and the adsorption behaviors of Eu^3+^ and Lu^3+^ satisfy the Freundlich isotherm model under the condition of a low concentration. This means that Eu^3+^ and Lu^3+^ will preferentially occupy stronger-binding sites, whereas at a high concentration, the adsorption behaviors of Eu^3+^ and Lu^3+^ satisfy the Langmuir isothermal model. The Langmuir model can describe chemisorption (chemical adsorption with about 50% coverage fraction) and physical adsorption is represented by the Freundlich model [34]. However, isothermal adsorption models can only approximately determine adsorption mechanisms, especially the Freundlich model, which is an empirical model and lacks specific physical significance; therefore, it is difficult to clarify the adsorption mechanism of Eu^3+^ and Lu^3+^ by model fitting [34]. The adsorption mechanism needs to be further corroborated by molecular dynamics simulations.

### 2.2. MD Calculation

#### 2.2.1. Adsorption Behavior of Eu^3+^ and Lu^3+^

In order to clearly reveal the difference in desorption characteristics between Eu^3+^ and Lu^3+^ on a deprotonated kaolinite (001) surface on a molecular scale, MD calculations, which can determine the statistical properties of a large system, were adopted.

**Figure 6:**
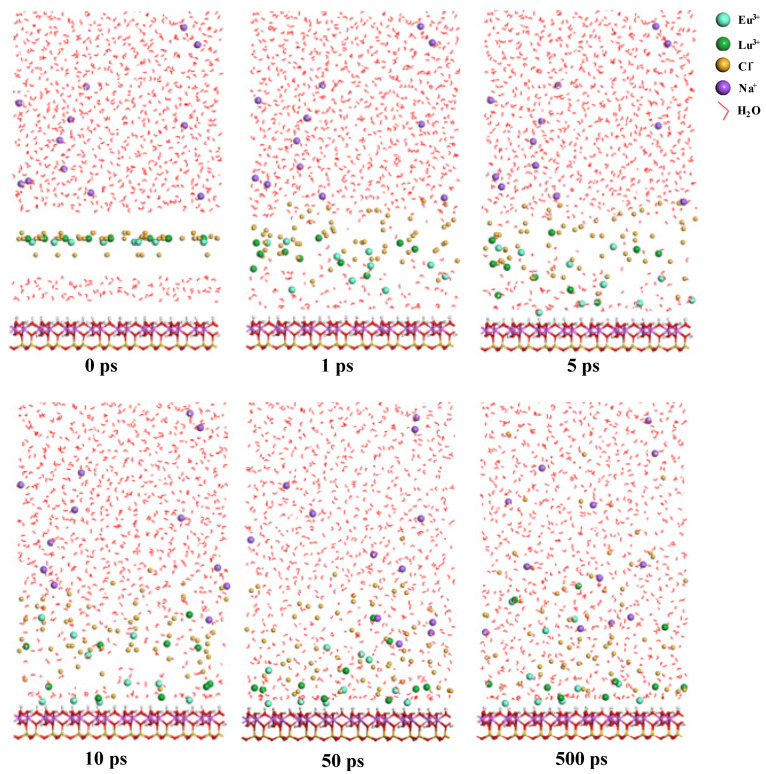
The adsorption geometries of Eu^3+^ and Lu^3+^ on deprotonated kaolinite (001) surface at different times.

The adsorption geometries of Eu^3+^ and Lu^3+^ on the deprotonated kaolinite (001) surface at different simulation times are shown in Figure 6. It can be observed that most Eu^3+^ and Lu^3+^ moved towards the kaolinite surface over time, forming an electric double layer. This surface is negatively charged, which is attractive to cations (Eu^3+^ and Lu^3+^). From the snapshots of 1 ps, 5 ps, and 10 ps, it can be seen that Eu^3+^ moved towards the surface significantly faster than Lu^3+^. The positions of Eu^3+^ and Lu^3+^ near the kaolinite surface remained almost unchanged at 50 and 500 ps, suggesting that a simulation of 500 ps was enough to reach adsorption equilibrium. When the system was at equilibrium, there were four Eu^3+^ ions on the deprotonated oxygen of kaolinite surface at distances of 2.14 Å, 2.21 Å, 2.45 Å, and 2.63 Å. These distances indicate that coordination bonds formed between Eu^3+^ and the surface. However, the smallest distance between Lu^3+^ and the deprotonated oxygen of kaolinite surface was 3.2 Å. When the distance between RE^3+^ and the kaolinite surface is less than 3 Å, it is called inner-layer adsorption, and when the distance is greater than 3 Å, it is called outer-layer adsorption. The MD results indicate that the interaction force between Eu^3+^ and the kaolinite surface was stronger than that of Lu^3+^.

The MD simulation explain for the difference in desorption behavior between Eu^3+^ and Lu^3+^ on the deprotonated kaolinite (001) surface. At a pH of 6, part of the hydroxyl surface is deprotonated and kaolinite mixes with the neodymium chloride and lutetium chloride solution, which contains active sites for inner-layer adsorption. In addition, the diffusion rate of Eu^3+^ on the deprotonated kaolinite surface was significantly faster than that of Lu^3+^. Hence, Eu^3+^ ions preferentially occupy the limited inner-layer adsorption sites, and these Eu^3+^ ions were difficult to desorb via ion exchange. Due to the limited inner-layer adsorption sites, Lu^3+^ can only be adsorbed via outer-layer adsorption. Therefore, the desorption rate of Eu^3+^ from kaolinite decreases significantly with an increase in pH, while the desorption rate of Lu^3+^ was less affected by the pH value.

#### 2.2.2. Concentration Distribution of Eu^3+^ and Lu^3+^ on the Surface

**Figure 7:**
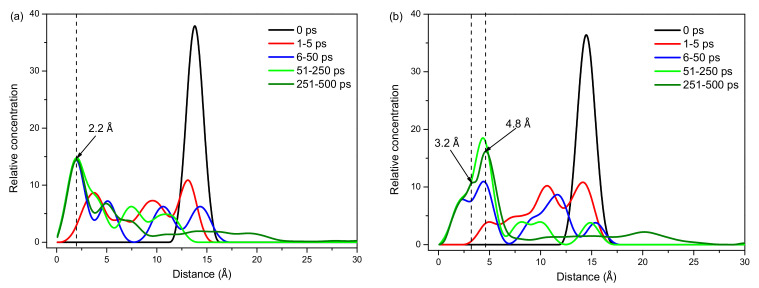
The concentration distribution profiles of Eu^3+^ (**a**) and Lu^3+^ (**b**) on the normal direction of kaolinite (001) surface at different time.

To quantify the adsorption of Eu^3+^ and Lu^3+^ on the kaolinite surface, the concentration profiles of Eu^3+^ and Lu^3+^ on the normal direction of the kaolinite (001) surface at different times are shown in Figure 7. As the simulation time increases, the peaks in the concentration profiles of Eu^3+^ and Lu^3+^ move towards the kaolinite surface. When the simulation duration is larger than 50 ps, the first peak of the concentration profile of Eu^3+^ basically coincides with different time periods, indicating that the position of Eu^3+^ near the surface of kaolinite remains unchanged after 50 ps, which was also verified by the adsorption geometries of Eu^3+^ at different times (Figure 6). The intensity of the first peak of the concentration profile of Lu^3+^ was still not the same after 50 ps, because there were no interactions between Lu^3+^ and the kaolinite surface; thus, Lu^3+^ can move freely in the solution.

The relative concentration profiles shown that the first concentration peak of Eu^3+^ appeared at 2.2 Å, indicating that a coordination bond was formed between Eu^3+^ and the kaolinite surface, whereas the distances between other Eu^3+^ ions and the kaolinite surface were larger than 3 Å, implying that only a some of the Eu^3+^ ions were adsorbed on the kaolinite surface by a coordination bond. However, the first concentration peak of Lu^3+^ appeared at 3.2 Å, while most of the Lu^3+^ appeared at 4.8 Å, indicating a more tightened adsorption of Eu^3+^ on the kaolinite. According to the literature [42,43,44], the density peaks located at a distance of less than 0.3 nm from the surface are linked to inner-sphere adsorption. Therefore, Eu^3+^ exhibited inner-sphere adsorption on the kaolinite surface through ligand bonding, while Lu^3+^ exhibited outer-sphere adsorption mainly through electrostatic interactions.

#### 2.2.3. Diffusion Abilities of Eu^3+^ and Lu^3+^

**Figure 8:**
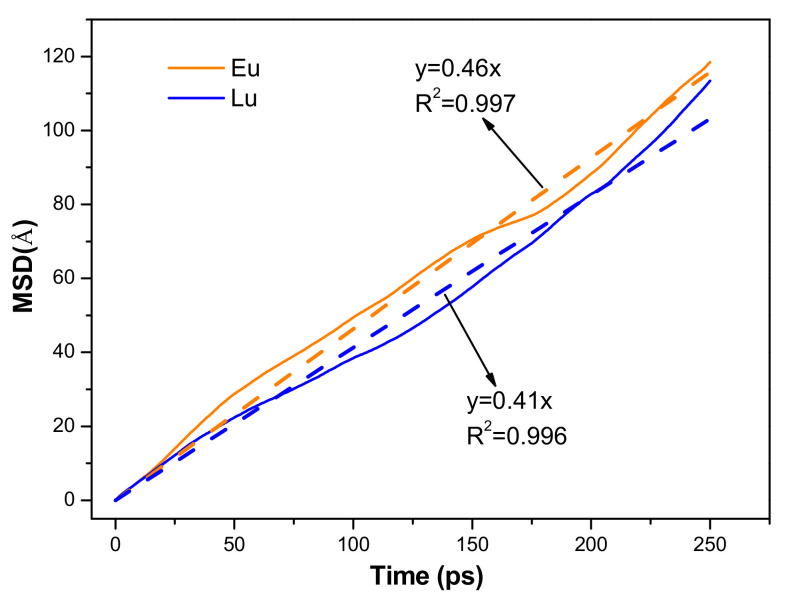
The mean squared displacement and linear fittings of Eu^3+^ and Lu^3+^ on kaolinite surface.

The MSD is linear with time and the statistics decrease with time; large fluctuations are often observed at the end. Therefore, the number of frames used in the calculation of MSD was no more than half of the total number of frames. The MSD of Eu^3+^ and Lu^3+^ at 0–250 ps was determined to investigate the diffusion ability. Figure 8 shows the mean squared displacement and linear fittings of Eu^3+^ and Lu^3+^ on the kaolinite surface. The self-diffusion coefficients (D) of Eu^3+^ and Lu^3+^ according to the fitting calculation are listed in Table 3. It can be seen from Table 3 that the self-diffusion coefficient of Eu^3+^ is 7.6 × 10^−9^ m^2^/s, which is larger than that of Lu^3+^, implying that the diffusion ability of Eu^3+^ was greater than that of Lu^3+^ in the simulation system. These results indicate that the adsorption of Eu^3+^ on the deprotonated kaolinite surface is kinetically dominant.

#### 2.2.4. Radial Distribution Function of Eu^3+^ and Lu^3+^

The RDF was used to study the aggregation of Eu^3+^ and Lu^3+^ with surrounding particles in order to obtain the coordination number. Generally, water molecules within 3 Å from the metal ion is regarded as the first hydration number. The intensity of the first peak of the atomic radial distribution function in RE-O_W_ (oxygen of water) indicates the ordering degree of molecular water around RE^3+^.

**Figure 9:**
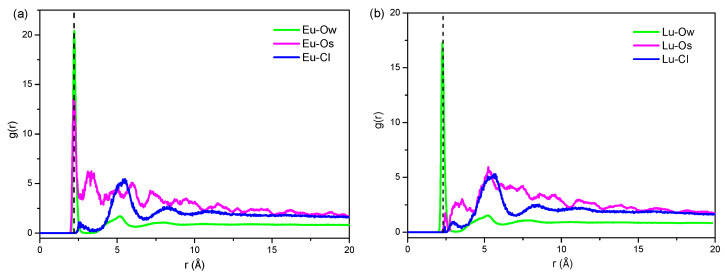
Radial distribution function of Eu^3+^ (**a**) and Lu^3+^ (**b**).

Figure 9 shows RDFs of Eu-O_w_, Eu-Os, Eu-Cl, Lu-O_w_, Lu-Os, and Lu-Cl pairs at 500 ps. It was found that the first sharp peaks of Eu-O_w_ and Lu-O_w_ are present at 2.23 Å and 2.31 Å, respectively, and the intensity of these peaks for Eu-O_w_ was higher than that of Lu-O_w_, implying that the ordering degree of water molecules around Eu^3+^ is higher than that of Lu^3+^. On the other hand, the higher ordering degree of water molecules is beneficial for the movement of Eu^3+^ to the kaolinite surface and the preferential occupation of adsorption sites. As seen in Figure 9, a strong peak appeared in the radial distribution function between Eu and O_s_ within 0–3 Å, while it did not appear in Lu-O_s_, indicating that Eu^3+^ is closer to the kaolinite surface. As for Cl^−^, there was no peak within 0–3 Å, with the first peak appearing around 5.17 Å, indicating that Cl^−^ ions were far away from Eu^3+^ and Lu^3+^, as EuCl_3_ and LuCl_3_ are fully ionized in aqueous solutions.

The coordination numbers at 500 ps were obtained by integrating RDFs of Eu-O_w_, Eu-O_s_, Eu-Cl, Lu-O_w_, Lu-O_s_, and Lu-Cl (Table 4). The first hydration number of Eu^3+^ was 7.1, which is less than that of Lu^3+^, due to the coordination number of O_s_ between Eu-O_s_ being higher than that of Lu-O_s_, which indicates that a certain amount of water coordinated to Eu^3+^ was replaced by O_s_. The coordination number of Cl^−^ between Eu-Cl and Lu-Cl was only 0.01, indicating that almost no Cl^−^ enters the first coordination layer of RE ions. The total coordination numbers of Eu^3+^ and Lu^3+^ are 8.12 and 8.01, respectively, which is close to the results reported by D’Angeol [45].

## 3. Experiments and Models

### 3.1. Experiment Details

#### 3.1.1. Experimental Materials

The raw kaolinite used in this study was provided by Kaolin Co., Ltd., (Suzhou, China). The other chemicals used, such as europium chloride, lutecium chloride, and ammonium sulfate, were obtained from Sinopharm Chemical Reagent Co., Ltd., (Chengdu, China).

#### 3.1.2. Desorption of RE Ions from Kaolinite

The mineralizing process of ion-adsorption RE ores was carried out in a slightly acidic environment; hence, kaolinite was added into a mixed solution of EuCl_3_ and LuCl_3_ at pH 6 in a mechanical shaker for 240 min. The adsorbents for Eu^3+^ and Lu^3+^ were desorbed using a 0.11 mol/L (NH_4_)_2_SO_4_ solution in the pH range of 2.5 to 6 for 240 min with a liquid–solid ratio of 50:1. H_2_SO_4_ was used to control the pH. After desorption, the rare-earth ion supernatant was obtained using centrifugation and filtration, and then, the concentration of rare earth ions in the supernatant was detected using an inductively coupled plasma–optical emission spectrometer (ICP-OES). In contrast, the same desorption process was also performed on raw kaolinite to ensure that the release Eu^3+^ and Lu^3+^ came from adsorption.

The leaching efficiencies were calculated using Equation (6).

(6) $$Recovery\left(\%\right)=\frac{c\;V}{m\;q_{e}}\times 100$$

where *c* is the concentration of RE ions that are desorbed in the leaching solution; *V* is the volume of the leaching solution; *m* is the mass of an adsorbent; and *q_e_* is the adsorption capacity of RE ions.

#### 3.1.3. Effect of Contact Time on Adsorption

The effect of contact time on the adsorption capacities (*q_e_*) of Eu^3+^ and Lu^3+^ on kaolinite was determined at a fixed pH of 6 at 25 °C. Furthermore, 1 g kaolinite was mixed with 25 mL of a mixed Eu^3+^ and Lu^3+^ solution with the concentration of 0.7 mmol/L for each REE. After adsorption, kaolinite was separated from the solution through centrifugation. After that, ultrapure water was used to remove the residue on the surface of the kaolinite and the kaolinite was then dissolved using acid to determine the *q_e_* of Eu^3+^ and Lu^3+^ on kaolinite via ICP-OES.

The equilibrium adsorption capacities (*q_e_*) were calculated by using the following equation:

(7) $$q_{e}=\frac{m}{M}$$

where *q_e_* is the adsorption capacity (mg/g) and m and M represent the masses of the adsorbed REE and the adsorbent, respectively.

#### 3.1.4. Adsorption Isotherm Experiments

The influence of the initial concentration of Eu^3+^ and Lu^3+^ on *q_e_* was studied by varying these concentrations in the range of 0.1–0.9 mmol/L with a contact time of 240 min at a fixed pH of 6.

### 3.2. Details of MD Calculations

Kaolinite is cleaved along the (001), (010), and (110) surfaces during crushing, with the basal surface (001) being the most easily cleaved, and thus this surface accounts for the largest percentage of the total area of kaolinite particles and has the greatest influence on the adsorption behavior of kaolinite [46]. Therefore, most of the current mechanistic studies have focused on the kaolinite (001) surface [44,47,48,49]. MD simulations were carried out using the Forcite module in Materials Studio software, which was developed by Accelrys Software Inc. Geometry optimization was carried out with a maximum iteration of 5000 in order to reduce the energy of the model to an acceptable level. Finally, a 500 ps MD simulation was performed at 298 K under an NVE ensemble while using the Universal [50] force field. Equations of motion were integrated with a time step of 1 fs for all dynamic runs, and the Nose function was selected for temperature control. The long-range electrostatic and van der Waals interactions were calculated using the Ewald- and Atom-based methods, respectively, with a cutoff radius of 12.5 Å. These parameters have been widely used to study the adsorption of metal ions on the kaolinite (001) surface [48,49].

**Figure 10:**
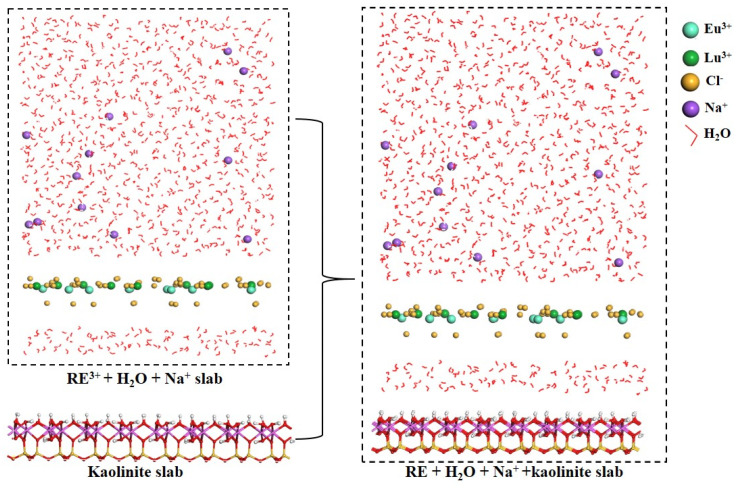
Initial adsorption model of EuCl_3_ and LuCl_3_ on deprotonated kaolinite (001) surface.

In our previous work [11], we found that the Al-OH on the kaolinite (001) surface was deprotonated when the solution pH was higher than 4.1; therefore, we only investigated the competitive adsorption of Eu^3+^ and Lu^3+^ on the deprotonated Al-OH surface. Otherwise, adsorption experiments imply that the number of deprotonated sites is limiting; hence, we assumed that 10% (three OH^−^ groups) of the surface hydroxyls were deprotonated and, in turn, the deprotonated surface was geometry optimized with the Castep module. Afterwards, the deprotonated surface was replicated two times in the x- and y-directions to obtain a super-cell with the following dimensions: 20.61 × 35.77 × 10.31 Å^3^ (Figure S1). In order to maintain the electrical neutrality of the system, 12 Na^+^ ions were added as balancing ions. The construction function in the Amorphous Cell module of Materials Studio was used to construct the following three solvent models: (1) 100 H_2_O molecules; (2) 10 Eu^3+^, 10 Lu^3+^ and 60 Cl^−^ ions; and (3) 900 H_2_O molecules and 12 Na^+^ ions. Then, the kaolinite model and three solvent models were combined using the Build Layers tool to obtain the initial adsorption model. A flexible simple point charge (SPC) model [51] was used for water molecules, while the Mulliken atomic partial charges of water molecules and RE^3+^ were used to determine the Castep. A vacuum layer with a thickness of 20 Å was added to the water molecule layer to avoid interference from the periodic image layer in the z-axis direction. The method of constructing the kaolinite/water/RE interface models is presented in Figure 10.

## 4. Conclusions

Adsorption experiments and molecular dynamics simulations were applied to investigate the competitive adsorption of Eu^3+^ and Lu^3+^ on kaolinite to illustrate why Eu^3+^ ions exhibit stronger pH-dependent desorption behavior. The adsorption of Lu^3+^ reached equilibrium in 60 min, while Eu^3+^ adsorption reached equilibrium in 120 min; however, both fit well with the pseudo-second-order model. Although the adsorption of Eu^3+^ and Lu^3+^ fit well with the Langmuir model, the R^2^ value of the Freundlich model for Eu^3+^ was high at 0.98, indicating that Eu^3+^ might be adsorbed at sites of a different energy on the kaolinite surface.

MD simulations showed that some of the Eu^3+^ ions move rapidly to the deprotonated kaolinite surface and occupy the limited inner-layer adsorption sites. This was indicated by the higher ordering degree of water molecules around Eu^3+^ compared to that of Lu^3+^, which was beneficial to the diffusion of Eu^3+^ to the kaolinite surface. Hence, the self-diffusion coefficient of Eu^3+^ is larger than that of Lu^3+^. As a result, some of the Eu^3+^ adsorbed to the kaolinite surface via inner-layer adsorption, and can be desorbed by a strong acid, while Lu^3+^ adsorbs via outer-layer adsorption, which can be desorbed via ion exchange. The different desorption behaviors of Eu^3+^ and Lu^3+^ were clarified by MD simulations on an atomic scale.

## Figures and Tables

**Table 1:** Kinetics parameters for the adsorption of Eu^3+^ and Lu^3+^ onto kaolinite.

RE	Pseudo-First-Order			Pseudo-Second-Order		
	*q_e_* (mg/g)	*k_1_* (min^−1^)	R^2^	*q_e_* (mg/g)	*k_2_* (g/mg/min)	R^2^
EuLu	0.28 0.172	0.024 0.022	0.978 0.954	1.078 1.281	0.299 0.685	0.998 0.999

**Table 2:** Constants for equilibrium isotherm models for Eu^3+^ and Lu^3+^ onto kaolinite.

Adsorption Isotherm Model	Unit	RE	
		Eu	Lu
Q_max-exp_	mg/g	1.12	1.32
1. Langmuir model			
*Q_L_*	mg/g	1.07 ± 0.04	1.28 ± 0.03
*K_L_*	L/mg	0.43 ± 0.12	0.45 ± 0.09
adj-R^2^	-	0.90377	0.94012
red-χ^2^	-	0.00493	0.00354
2. Freundlich model			
*K_F_*	${\left(mg/g)/(mg/L\right)}^{n_{F}}$	0.49 ± 0.03	0.64 ± 0.06
*n_F_*	^−^	0.19 ± 0.02	0.15 ± 0.02
adj-R^2^	^−^	0.97506	0.93089
red-χ^2^	^−^	0.00128	0.00408
3. Langmuir-Freundlich model			
*Q_LF_*	mg/g	1.56 ± 0.24	1.49 ± 0.14
*K_LF_*	L/mg	0.09 ± 0.08	0.30 ± 0.12
*n_LF_*	-	0.41 ± 0.08	0.54 ± 0.13
adj-R^2^	-	0.99157	0.97841
red-χ^2^	-	4.32 × 10^−4^	0.00128

**Table 3:** Diffusion coefficient values of Eu^3+^ and Lu^3+^.

Ion	Eu^3+^	Lu^3+^
*D* (m^2^/s)	7.6 × 10^−10^	6.8 × 10^−10^

**Table 4:** The coordination parameters of Eu-O_w_, Eu-Cl, Eu-O_s_, Lu-O_w_, Lu-Cl and Lu-O_s_.

	Start Radius (Å)	First Sharp Peak (Å)	Cutoff Radius (Å)	CN
Eu-O_w_	2.01	2.23	3	7.1
Eu-Os	1.95	2.17	3	1.01
Eu-Cl	2.49	2.93	3	0.01
Lu-O_w_	1.97	2.31	3	7.9
Lu-Os	2.41	2.47	3	0.1
Lu-Cl	2.41	2.93	3	0.01

## Data Availability

Data are contained within the article.

## Supplementary Materials

The following supporting information can be downloaded at: https://www.mdpi.com/article/10.3390/molecules30040838/s1, Figure S1: The modeling process of kaolinite super-cell; Figure S2: The concentration distribution profiles of H_2_O on the normal direction of kaolinite surface.

## Abbreviations

The following abbreviations are used in this manuscript:

RE	Rare earth
MD	Molecular dynamics
DFT	Density functional theory
MSD	Mean square displacement
