# Supplementary material for: Theoretical Investigation of Competitive Adsorption of Light and Heavy Rare Earth Ions on the (001) Surface of Kaolinite
Source: Molecules. 2025 Feb 11;30(4):838. doi: 10.3390/molecules30040838 (PMC11858435; doi:10.3390/molecules30040838)
Supplement: Supplementary file 1 [file molecules-30-00838-s001.zip › molecules-3427232-supplementary.pdf]

Supporting information for

Theoretical investigation of competitive adsorption for light and heavy rare earth ions on kaolinite (001) surface

Sen Qiu [1,2], Yijin Hua [1], Zehao Fan [3], Qibang Long [3], Kuifang Zhang[1, 2], Xuwei Lian [1], Tao Tu [1], Li Li [1], Tingsheng Qiu [3*]

1 Collge of Rare Earth and New Materials Engineering, Gannan University of Science and Technology, Ganzhou 341000, China.

2. Funded by Key Laboratory of Ionic Rare Earth Resources and Environment，Ministry of Natural Resources of the People's Republic of China

3 College of Resource and Environmental Engineering, Jiangxi University of Science and Technology, Ganzhou 341000, China.

* Corresponding authors.

Email addresses: 9019810001@jxust.edu.cn (T. S. Qiu).

The modeling process of kaolinite super-cell

In order to obtain the deprotonated kaolinite super-cell, the geometry structure of kaolinite unite cell (Figure S1a) was optimized in Castep module, after that it was expanded into 2×2 super-cell (Figure S1b), and then 10% (3 OH^- group) of surface hydroxyl in 2×2 kaolinite super-cell were deprotonated and the deprotonated surface was geometry optimization with Castep module (Figure S1c). To simulate larger system, the deprotonated surface of kaolinite 2×2 super-cell was replicated 2 times in x-direction and y-direction to obtain a super-cell with the following dimensions 20.61 × 35.77 × 10.31 Å^3.

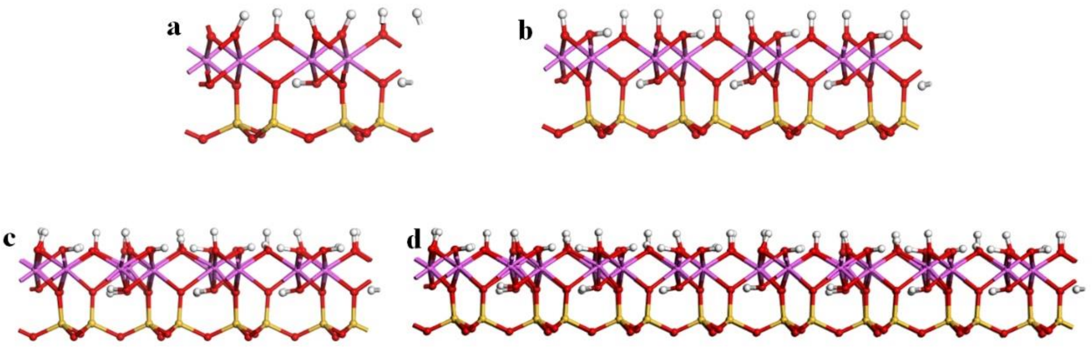

Figure S1. The modeling process of kaolinite super-cell

Concentration distribution of H_2O on the surface

Figure S2 shown the concentration profiles of H_2O on the normal direction of kaolinite (001) surface. The first peaks of H_w (H atom of water) appeared at 2.9 Å, which is closer to the surface than O_w (O atom of water), indicating that the hydrogen atoms of the water molecules are towards the kaolinite surface, because the O_w tends to form hydrogen bonds with the kaolinite surface. With the increase of distance between water molecules and kaolinite surface, the order degree of water molecules decreases gradually. The results also suggesting that the hydrophily of kaolinite surface is strong.

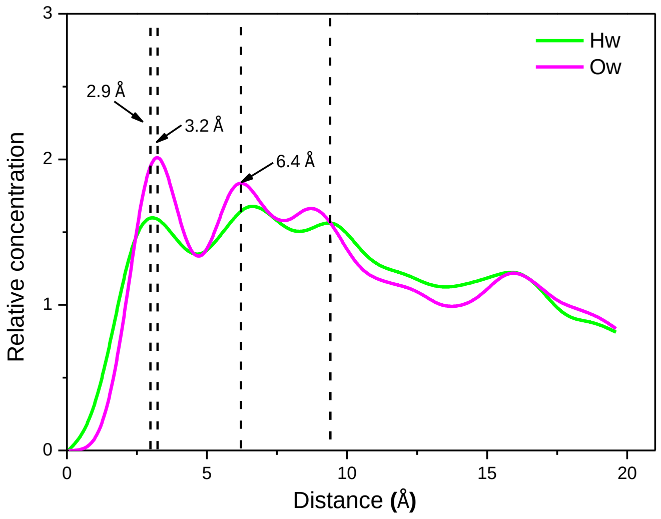

Figure S2. The concentration distribution profiles of H_2O on the normal direction of kaolinite surface
